# Abstracts and Gleanings

**Published:** 1882-12-20

**Authors:** 


### ABSTRACTS AND GLEANINGS.

**Skin Grafting.**—The patient, a pretty little girl of eight, was admitted into the Wellington ward of St. George's hospital with the history that two years ago previously her dress had caught fire, burning both legs from the hips to the knees severely. After a year's treatment the left thigh had healed up; but the right had never gotten better, and presented a terrible ulcer, extending all down the outer side. She was a bright, intelligent little thing, and her sad condition excited much sympathetic interest. For four months she lay there without any signs of improvement. Though nourishing food, with wine and strengthening medicines, was freely administered, and all manner of local remedies applied, particularly that most excellent dressing, carded oakum, all was in vain; and when, on the 5th of May, the child was brought into the operating theatre, and placed under the influence of chloroform, it certainly appeared to us to be as unlikely a case to afford a fair criterion of a new treatment as could well be imagined. Two small pieces of skin were then snipped from the back with a pair of sharp-pointed scissors, and then imbedded—planted, in fact—in the granulations or "proud flesh" of the wound—two tiny atoms, scarcely bigger than a pin's head, and consisting of little more than the cuticle or outer skin which we raise in blisters by rowing or exposure to a hot sun. Five days later no change was visible; and by and by the operation was considered to have failed, since the pieces of skin had disappeared, instead of growing as had been expected. But twelve days after the operation two little white cicatrices appeared where the seed had been sown; and in my notes I find that a week later these were big enough to be dignified as "islands of new tissue." The most wonderful part of it is that not only did these islands grow and increase rapidly in circumference, but the fact of their presence seemed to stimulate the ulcer itself, which forthwith took on a healing action around its margin. Several more grafts were implanted subsequently, including morsels from Mr. Pollock's arm, from my own and from the shoulder of a negro; the last producing a white scar-tissue like the rest. In two months the wound was healed, and the little patient discharged cured.

Skin-grafting is now performed daily in surgical practice, and a special instrument—a combination knife and scissors—has been invented for the purpose. It is impossible to estimate the immense benefit of this discovery to mankind in many different aspects. Poor people, hitherto incapacitated from labor by "incurable" ulcers, and for many years a burden on their parish, or inmates of workhouses and asylums, will now again resume their place in the great toiling hive, from whose daily labor is distilled the prosperity of a nation. Von Graefe's operation of iredectomy, where by hundreds of people, who were formerly considered irremediably blind, are now restored to sight by a simple proceeding, is said to have exercised a very appreciable effect on the poor-rates of the country. As an instance of true transplanting, John Hunter's celebrated experiment of causing a human tooth to take root and grow in the comb of a cock is a well-known instance. Dentists now-a-days remove teeth, and having excised diseased portions, replant them in their sockets with frequent, though not invariable, success; and cruel plastic operations have been performed on rats, by which they have been joined like Siamese twins, or their tails caused to grow from their shoulders, or between their eyes. The late Mr. Frank Buckland, in his "Curiosities of Natural History," gives an amusing account of an action at law brought by M. Triguel, a French naturalist, against a zouave who had sold him what was termed a "trumpet-rat" for 100 francs; the said trumpet-rat proving to be an ordinary "varment," with the tip of another rat's tail planted in its nose, and growing there.—*Medical Gazette.*

**Vesico-Vaginal Fistula Cured by Position.**—The following case came under my observation in Fluvanna county, Va., while associated with my father, Dr. R. J. Winn:

On the 23d of January, 1881, he was called to see Mary B., colored, æt, 18, suffering from incontinence of urine and extensive excoriation of the genitals therefrom, supervening upon the birth of a large still-born child, at full term, four days before.

She was not attended by any physician in this, her first confinement; consequently the history of the labor must necessarily be meagre. The fact was obtained from the mother, however, that the labor was tedious, lasting 48 hours. (Her mother is a monthly nurse, and was the officiating accoucheur.) Whether her statements be correct or not, this much was plainly evident, viz.: a vesico-vaginal fistula detected, both by digital and speculum examination.

The patient was informed of the ultimate necessity of an operation after involution had occurred, and she had obtained some relief from the scalding urine. As a means of affording temporary relief from the trouble, she was placed in the genu-pectoral position with instructions to remain thus as long as consistent with comfort, thus enabling the urine to collect in the fundus of the bladder. No catheter was introduced, but she was directed to change her position at intervals of three or four hours, and let the contents of the viscus pass away.

A moderately strong solution of bi-carbonate of soda was ordered to be given as a vaginal enema immediately after each urination, as also a vaginal enema of carbolic acid solution, three times daily.

With these general directions she was left with instructions that her father must report in eight or ten days, reporting the progress of the case. Failing to receive any tidings of the case, a verbal request was sent to know why such report had not been made. Whereupon, on the 12th day of February (just twenty days from beginning of treatment) he reported that his daughter was well.

To satisfy ourselves of the truth of the old man's statement, we made a special visit on the 14th of February, and to our gratification and no little surprise, we found a firm, smooth cicatrix taking the place of the fistula, which three weeks before gave every indication for the necessity of surgical aid.

No claim is made here for originality, for similar results have been obtained in the hands of other practitioners. Yet the facts named prove the value of position in the management of vesico vaginal fistula, of recent origin, and warrant its fair trial bfore resorting to the usual operations.—Dr. J. F. Winn, in the *Virginia Medical Monthly.—Detroit Clinic.*

**Sensible.**—In these days, when so many doctors may be found who are little better than professional loafers, so many who discourage the reading of medical works who express their contempt for original research and scoff at medical journals, regarding the accumulation of money as the only test of professional success, and who depend on their own personal shrewdness and the gullibility of the people at large to excuse the title under which they thrive, the following, relative to the life of Dr. Geo B. Winston, from the St. Louis Courier of Medicine, is refreshing:

A friend once remarked to him, "Doctor, what necessity is there for this ceaseless labor and study at your time of life?" With a look of astonishment never to be forgotten he replied, "My dear sir, I am under bonds to do it. When I offered my professional services to this community there was an implied covenant on my part that, so far as God gave me strength and ability, I would use them for gathering up and digesting all that has been said or written in regard to the diseases to which human flesh is heir; and if I should lose a patient because of my ignorance of the latest and best experience of others in the treatment of a given case, a just God would hold me responsible for the loss, through inexcusable ignorance, of a precious human life, and punish me accordingly; and whenever I get my consent to be content with present professional attainments, and trust my own personal experience for success, I will withdraw from practice and step from under a weight of honorable obligations which, with my best endeavors to meet them honestly and conscientiously, still sometimes is almost heavier than I can bear."—*Southern Med. News.*

**To Keep the Hypodermic Syringe in Order.**—Some months ago I noticed in some periodical, the statement that a few drops of glycerine placed in a hypodermic syringe would prevent, measurably, the shrinkage of the piston packing when the instrument was not in use. I suppose that many physicians have felt the need at times of having such instrument in immediate working order. I know that I had, until I accidentally discovered the good influence of glycerine in that respect. I have long been in the habit of drawing into the barrel each time after use, a small quantity of a mixture of one part of distilled water to three parts of glycerine, allowing it to remain until the instrument is wanted for use, with the advantage of having it always in a serviceable condition.—*Peoria Med. Monthly.*

**Treatment of Gonorrhœa by Injections of Sulphurous Acid Diluted with Water.**—For some time I have treated all cases of gonorrhœa with injections of sulphurous acid diluted with water, and as the results in my hands have been very satisfactory, I write in the hope that others may be induced to give this method a trial.

I do not offer any theory on the subject, I simply state the fact that I have now treated sixteen cases of gonorrhœa, using no other medicine, and they all returned to duty in an average of six days, I have not observed a relapse or any bad effect. The majority of the cases were second attacks, but those suffering from primary attacks of the disease recovered equally fast.

When I commenced this method of treatment, I used much stronger injections than I do at present. I find sulphurous acid one part to fifteen of water quite strong enough for most cases. The rules of treatment I recommend are: place the patient on low diet, and administer injections of sulphurous acid diluted in water one to fifteen, three times a day, no other treatment being necessary. I find it is necessary for the attendant to give the injections, for if it is done by the patient it is never well done, most of the fluid escaping back outside the nozzle of the syringe. The injection should be kept in the urethra from three to five minutes. If the patient complains of much pain, or if there is a tendency to chordee, it will then be sufficient to administer the injections once or twice in twenty-four hours.

If these instructions are strictly followed the purulent discharge will become scanty at the end of the first day, and on the third it will be replaced by a thin, gleety discharge, which also disappears in a couple of days. While this watery discharge lasts I usually administer only one injection daily. I find that the first injection frequently causes pain, which is not so much complained of afterwards. I, therefore, in a few cases give the first injection very much diluted—one in twenty, afterwards using one in fifteen. It is necessary to see that the sulphurous acid is fresh and good before it is diluted to the required strength.—*W. D. Wilson, M. B., in London Lancet.*

**Faith as an Element of Success in Medicine.**—The effect of the mind on the body is now recognized by all writers on therapeutics, and there can be no doubt that the patient's mind is often affected by what he sees his physician's to be. If the doctor evidently has thorough faith in the treatment he is pursuing, the patient is apt to be inspired with sympathetic confidence, and the treatment is then more likely to be successful. On the other hand, an evident lack of confidence on the part of the practitioner may cause a distrust in the sick man's mind which will perhaps interfere with the desired result. Dr. Fothergill, referring to this subject, says:

If the medical man speaks to the patient with doubtful accents and hesitating utterances, he does not inspire confidence; he really sows distrust. This is the explanation of the successful treatment of a case by one man where another has failed, the remedial measures being much the same. The one carries the patient with him to the restoration of health; the other intensifies a morbid state, and tends to make it permanent.

This is a matter too little thought about. Just as a weak-willed medical man fails to do certain patients good, and lack of decision of character unfits a medical man for dealing with emergencies, where the judgment must be prompt and the action energetic, so the therapeutic nihilist, who doubts the efficacy of drugs, and leaves the patient to nature, disheartens many patients, and leaves them chronic valetudinarians; while in the hands of an enthusiast the cases would soon move onward to a satisfactory termination. There are some men who are "doubting Thomases;" there are others who decry what they do not understand, and deprecate remedies with whose potency they are unacquainted, who do infinite, immeasurable harm to their patients. An eclipse of faith in medicines has now existed some time; but the darkness is beginning to move away, and a return of faith, stronger, firmer, more capable of giving a *raison d'etre* for its existence than in the past, is dawning—the daybreak of happier times for those who are stricken down with illness, or crippled in their working power by incapacity in their digestive viscera. This therapeutic nihilism is a passing wave of opinion, a temporary mental state, the end of which is at hand; and the sooner it is over the better for all. The patient's prospects will be all the brighter; the medical man all the happier for feeling that the patient has got some "value received" in return for his outlay. A healthier condition of thought on matters medical will generally obtain; for quacks, charletans and irregular practitioners of all kinds are to a great extent fostered by the recent want of faith in the medical profession. When a man is sick, what he wishes is to get well; the means to him is a matter of comparative indifference.—*Journal of Chemistry*.

**The Antiseptic Treatment of Typhoid Fever.**—At a meeting of the Societe Medicale des Hopitaux, June 9th, M. Ferrand presented the candidate's thesis of Professor Desplat, of Lille, upon the comparative action of carbolic acid and salicylate of soda. The views presented were that the above drugs were excellent antipyretic and antialgesic agents—sure, rapid, and permanent in their action, but, at the same time, easily eliminated, and, therefore, but slightly dangerous. Except in acute rheumatism, M. Desplats did did not find any marked difference in their action.

The discussion which ensued turned upon the use of carbolic acid in typhoid fever. Thirteen members took part and related their experience. Three or four did not commit themselves; the remainder agreed in saying that the drug in question, used as recommended, had a dangerous tendency to depress the system, and to produce pulmonary congestion, exhausting sweats, and albuminuria or polyuria.

It was unanimously voted that the use of carbolic acid in typhoid fever, when given as recommended (in half-gramme or gramme doses by enema twice a day), was dangerous, and without effect upon the course of the fever.

Dr. Ramonet, Physician-in-Chief at the Military Hospital of Boghar, in Algeria, has contributed an article upon the use of carbolic acid in typhoid fever, expressing directly contrary views to the above. He is a follower of Desplat, except that he uses smaller doses, generally not more than two grammes per diem, by injection. The effect, he says, is to lower the temperature nearly 4° F., and to produce a most favorable change in the progress and symptoms. He has treated forty-one cases, with a mortality of only two, or 4.9 per cent. The average mortality from this disease in the army is twenty-one per cent.

On August 22d, at the Academy of Medicine, M. Vulpian read a paper upon the use of salicylic acid in typhoid fever. M. Vulpian based his therapeutics upon the theory that there is a bacillus of enteric fever in the intestine, and that this bacillus ought to be ferreted out and killed with an antizymotic. Having tried iodoform, boric acid, phenate of soda, and salicylate of bismuth with no effect, he finally settled upon salicylic acid. This in daily doses of two or three grammes was ineffective, but in doses of six or seven grammes daily (gr. xl. to gr. l., every two hours!) most satisfactory results were obtained in a lowereng of the fever and a general amelioration of symptoms. M. Vulpian concluded that this drug, without being curative, had an undoubted modifying influence upon typhoid fever. He thought also that salicylic acid taken by the mouth might act as a prophylactic. The discussion which followed brought out very little. It was only evident that M. Vulpian's views were theoretical, and that the clinial tests of his reputed remedy were not at all conclusive. Salicylic acid has been tried in Germany and America with no very good results, as yet reported.—*N. Y. Med. Record.*

**Malaria in Skin Diseases—A Correction.**—Dr. Lunsford P. Yandell, of Louisville, says:

Some time since the following paragraph appeared in the Michigan *Medical News*, and has been widely copied in the medical journals of the country:

"A century ago John Hunter divided all skin diseases into three classes, one of which is cured by mercury and the iodides, a second by sulphur, and a third class which the devil himself can't cure. Dr. L. P. Yandell, who quotes Hunter as above, is given credit for a much less complex classification than even this. He attributes all skin eruptions to malaria. Quinine is a specific for malaria; ergo, quinine is the remedy for all skin eruptions."

Q. E. D.

The subjoined extracts are from a supplement to a report read to the American Dermatological Association, September, 1877. A copy of this report will be gladly sent to any one desiring it:

"From the criticisms which have been made on my views, I find that I have not succeeded in making myself perfectly understood. What I have contended for, and what I have reiterated, is simply this: Malaria is the chief source of acute skin disease. Scrofula is the chief source of chronic skin disease. The more inveterate cases of skin disease are often due to the co-existence of these two things. The specific exanthems, of course, are not included here, but I contend that their progress and termination are often largely influenced by the presence of malaria and struma. I do not claim that malaria and struma are the sole causes of the dermatoses. Indeed, many of the dermatoses may exist independently of malaria or struma, and most frequently some exciting cause is necessary to develop the cutaneous eruption. Among the exciting causes are irritants, injuries, insufficient or improper ingesta, vicissitudes of temperature, alcohol, dentition, menstruation, parturition, lactation, etc. The proofs of the truth of my views are, in the first place, that the diseases of the skin are cured more certainly and more quickly by the anti-malarial remedies on the one hand, and by the anti-strumous on the other, than can be done by any other line of therapeutics; and in the second place, that careful and painstaking investigation will, in the majority of dermatoses, make apparent the existence of the malaria or the struma, as the case may be.

"In conclusion, I desire to impress upon the reader that my views are not confined to the skin diseases. What produces disease here will produce it in all other organs of the body. What is true of dermatology is equally true of gynecology and ophthalmology and otology, and it is just as true of the diseases of all the other regions of the body."

Subsequent observation has confirmed my belief in the correctness of these views.

**Puncture and Aspiration in Intestinal Invagination.**—We read in Paris *Medical*, January 28th, 1882, that Dr. Godfrey has treated a case of intestinal invagination as follows: The patient, a man 37 years old, was vomiting a greenish-yellow liquid having a most offensive fecal odor. His abdomen was distended, and very tender to the touch; and distinct fluctuation together with dullness were perceptible over the entire course of the colon. The umbilical region was somewhat tympanitic. Great tenesmus existed, and the efforts at defecation only resulted in the passage of a little bloody mucus. Having carefully ascertained that no hernia existed, the intestine was punctured, first in the left, then again in the right iliac region, the largest needle of a Codman and Shurtleff aspirator being used for that purpose. More than a pint of liquid, similar to that vomited, was thus withdrawn, and the patient felt somewhat relieved. Vomiting now became less frequent, and finally ceased. Morphine, first hypodermically, then by the mouth, procured sleep, and after three days the intestine had resumed its functions, and in less than a week the patient was again well.—*Med. and Surg. Reporter.*

**Curing Disease by Anointing With Oil.**—The mountain evangelist, Rev. Mr. Barnes, having ousted Satan from a number of Western strongholds, has attacked Cincinnati. His campaign may attract medical attention, since he cures disease as well as saves souls. His method is to anoint with oil, at the same time uttering a brief prayer. A reporter, interviewing him, asked if he had cured many diseases in Indianapolis. "There were," he said, "some very remarkable cases. One was that of an old man, almost helpless with rheumatism, who was obliged constantly to use crutches. He was a member of the Roberts Park Methodist Episcopal Church. He was anointed with oil, and his faith took hold on Christ for healing, which occurred almost instantly. The next day he came to the church without crutches, and testified before the assembly of the healing, and declared he could run a foot-race, and praised the Lord with all his might. Another case was one of a woman subject to spasms for years, and many others." Here Mrs. Barnes declared it to be so common a thing in their experience that the wonder is, not that the persons are healed, but that all Christians do not at once go to Jesus when they are sick.—*N. Y. Med. Record.*

**Mustard in the Treatment of Smallpox.**—Dr. Lyndon (in *Medical and Surgical Reporter*) says: Just before the close of the war I was called to prescribe for a Confederate soldier, suffering with great nausea. A large mustard plaster was ordered to be placed over the stomach. A few hours afterwards my attention was directed to an eruption covering the part where the mustard had been placed. It was a well developed case of smallpox. There was no eruption on any other part of the body. The pustules were well developed, with the characteristic pit. I did not have another opportunity to try it, but believe a mustard plaster applied to any part of the body will bring out the eruption twenty-five to thirty-six hours earlier than usual, so that a diagnosis can be made on the first day of the fever. I believe it possible to invite all the eruptions to any part of the body, and thus avoid the pitting of the face. And in malignant cases, where the poison produces death before the eruption appears, the mustard might possibly bring out the eruption and save the patient. The experiment is easy and harmless.

**Treatment of Cerebro-Spinal Meningitis.**—Prof. H. C. Wood, in a clinical lecture in the Medical Gazette, sums up as follows: During the first three or four days in the strong and robust, leeches or cups may be applied to the temples or nape and upper part of the spine. Ice-bags are applied to the head and back of neck for the first days—in many for a week. To relieve headache, restlessness and dilirium, bromide of potash is the best agent, gr. 20 to 30 every three hours. Its efficacy is increased by adding chloral (ten grain doses usually) or in those who cannot take chloral, tinct. hyoscyami (drachm doses). It is advantageous to add also tincture of castor (drachm doses) in the hysterically inclined. If possible don't use opium, but sometimes it becomes necessary, as the remedies already named occasionally fail. The temperature is not apt to run over 104° (a very harmless height) in adults except at the close, and quinine is not indicated; moreover, it has no effect in lowering the temperature in this particular disease. The best way to lower temperature, if this be an object, is by cold affusion, cold and tepid baths, or the cold pack.—*Maryland Med. Journal.*

**How Parturition is Managed by the Beniamir Arabs and the Abyssinians.**—When a woman is in labor she is attended by some knowing old woman (they would rather die than let a man come near them.) Should the labor be protracted, a rope is put under each arm and attached to a piece of wood overhead. On this rope she presses each time she has a pain, and in this standing position she is delivered. I asked: "How do you manage, supposing the child is in such a position as to require instrumental interference?" "Well, then," he said, "we can do nothing, and she has to die." Should she suffer from flooding, she is put to sit in hot water for ten minutes, and then a bandage is wound around her several times as tightly as it can be put; a decoction is then given her to drink, made from tamarinds and the leaves of some tree, the name of which I was unable to ascertain; and if she lives she is not allowed to taste water for seven days, but has nothing but warm milk.

The Abyssinian mode of conducting labor is also curious. The woman lies upon her back, two stones are pushed under her buttocks, two women grasp her legs, and just as the child is entering the world a tray full of flour is put under to receive it.—*Medical News.*

**New Method of Reducing in Dislocation of the Humerus.**—Mr. James E. Kelly (*Dublin Journal of the Medical Sciences*) recommends the following as successful when other plans have failed: The patient should be placed as close as possible to the edge of the couch, on his back, with his head low. The operator places the injured arm at right angles with the body, and standing against it, with his side to the patient, and his hip pressed firmly but not roughly into the axilla, he folds the arm and hand of the patient, close around his pelvis, and fixes the hand firmly by pressing it against the crest of his ilium. The second stage, during which the reduction is effected, is very simple, consisting merely of a rotation or version of the surgeon's body with a force and rapidity which necessarily vary with the peculiarity of the dislocation—some yielding most readily to a sudden and most powerful effort, and others to gentle and gradually increasing traction.—*Chic. Med. Review.*

**Eczematous Ulcer of Leg.**—Dr. Willard Chaney, city physician, Detroit, has had excellent success in the treatment of this disease with ointment of petroleum and iodoform. A widow, 30 years of age, hard-working, living in damp and illy lighted apartments, had eczematous ulcer, large as a silver dollar, on the inner aspect of lower third of leg. Anorexia, anæmia, hysteria with copious sanious discharge from painful ulcer, were prominent symptoms. The doctor ordered her all day rides in the open air of the Detroit river ferries; to take internally, three times a day, 20 drops of muriated tincture of iron with one thirtieth of a grain of corrosive sublimate, in suitable vehicle; applied to ulcer daily: ℞ unguent petrolei ʒ ii., iodoformi ʒ j., M.; with firm even pressure by means of roller bandage.—*Med. Red.*

**Abdominal Deliveries in the United Siates for 1880.**—Dr. R. P. Harris, of Philadelphia, in his record of the abdominal deliveries in the United States, gives as the result for 1880 five Cesarean operations, and three Porro Cesarean. The results of the classic Cesarean sections are encouraging, three women and four children having been saved. The antiseptic method of Lister, the cleansing of the abdomen from blood and other fluids, the wire uterine-suture, and numerous minor modifications of the operation have undoubtedly contributed to this result. The uterus was sutured in three out of the five cases, with a saving of two. The Lister method was used in two cases, and phlegmasia dolens, which attacked two of the American Porro cases, occurred in one case.

The results of the three Porro Cesarean sections were one woman and two children saved. With enlarged experience and improved methods we should equal the record of the maternities of Milan and Vienna. The Santa Caterina of Milan has saved six women out of eight, and the Krankenhaus of Vienna eight out of eleven; the last six operations in each having been successful.—*Boston Med. Journal.*

**Chloroforming Mice.**—A French Surgeon says that on chloroforming some mice and lifting them by their tails, they tri edto bite, but on laying them again in a horizontal position, they resumed insensibility. Acting on this hint, when a patient showed signs of a collapse under a dose of chloroform, he dropped the patient's head over the bedside and raised the feet quite high. The patient at once became conscious; when laid straight on the bed he became insensible again, and a return to lowering the head and raising the feet for ten minutes was required to fully counteract the chloroform. It is thought, that by aid of this treatment, anæsthetics may be used with a high degree of safety.—*Ind. Prac.*

**Transplantation of Muscle from the Dog to Man.**—After the removal of a large fibro-sarcoma from the biceps of a woman, æt. 36, Helferich filled the gap with a freshly-cut piece of muscle from a dog, fastening it with 6 lower and 30 upper catgut ligatures. Cure followed antiseptic dressing. The patient can readily flex and extend the arm. Electrical examination by Ziemssen showed no abnormality and the transplanted muscle seems to have retained its vital functions.—*Berl. Klin. Wock.*, No. 26 and *Cin. Lanc. and Clinic.*

**Extraction of Teeth During Pregnancy.**—Dr. Chester (in *Southern Practitioner*) says: I will inform you that, in June, 1878, Mrs. R. W. G., 22 years, was delivered by me of a 7-months child, who lived only a few hours. In August, 1879, I again delivered her of a 6-months child, who was born dead. In January, 1881, she was in pregnancy six months; was suffering intensely from toothache. I decided to extract two grinders; in April, '81, I delivered her successfully of a full term child. The extracting of the teeth having caused great relief without bad effect.

